# A Novel Intensity-Modulated Radiation Therapy Approach to Reduce Tongue Radiation Dose Using the BinkieRT Intraoral Device: A Case Report

**DOI:** 10.7759/cureus.98604

**Published:** 2025-12-06

**Authors:** Kanta Shiojima, Seiya Isozaki, Toshiaki Matsui, Takumi Kodama, Shigehiro Kudo

**Affiliations:** 1 Radiation Oncology, Saitama Cancer Center, Saitama, JPN

**Keywords:** binkiert, imrt, intensity-modulated radiation therapy, intraoral positioning device, oral cancer, oral mucositis prevention

## Abstract

Radiation therapy plays a pivotal role in the management of oral cancer. However, radiation-induced mucositis and dysgeusia frequently impair patients’ quality of life. To mitigate these risks, intraoral positioning devices have been introduced to minimize radiation exposure to the oral organs at risk. We report a case of recurrent buccal mucosa cancer treated with intensity-modulated radiation therapy (IMRT) using a commercial intraoral device, BinkieRT (Paprica Lab., Seoul, Republic of Korea). The device displaced the tongue contralaterally and separated the oral cavity from the target volume, leading to a reduction in radiation dose to the tongue and oral mucosa without compromising target coverage. Compared with handmade spacers or bite blocks, BinkieRT offers distinct advantages in terms of ease of use, hygiene, and reproducibility. The observed immediate dosimetric improvement indicates the potential to reduce the incidence and severity of radiation-induced oral toxicity. Its noninvasive and patient-friendly design also makes it applicable to a wide range of clinical situations, including re-irradiation cases or elderly patients with limited tolerance. BinkieRT provides a simple and practical approach to reduce unnecessary radiation exposure in oral cancer IMRT. Further clinical studies are warranted to validate its efficacy and establish optimal usage protocols.

## Introduction

Radiation therapy (RT) plays an important role in the management of head and neck cancers, including oral cancer. However, radiation-related adverse events (e.g., oral mucositis, dysphagia, dysgeusia, and xerostomia) remain major clinical concerns. Studies have shown that increased radiation dose to the oral cavity, including the tongue, is associated with the incidence and severity of these adverse events [1-3]. To mitigate these risks, several intraoral devices have been developed to reposition the tongue and spare surrounding healthy tissues [4-6]. This case report describes the dosimetric benefits of using the commercial intraoral positioning device BinkieRT (Paprica Lab., Seoul, Republic of Korea) during intensity-modulated radiation therapy (IMRT) in a patient with recurrent buccal mucosa cancer. We compared dose-volume histograms (DVHs) of the tongue obtained with and without the device to verify reductions in radiation exposure to critical structures. To the best of our knowledge, no previous studies have compared tongue-body DVHs with and without the use of BinkieRT.

## Case presentation

**Figure 1:**
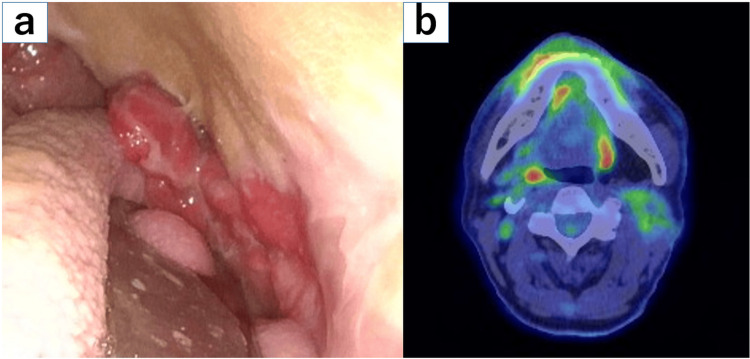
Local recurrence at the inferior margin of the flap after surgery for left buccal mucosa carcinoma. (a) A flexible nasopharyngeal laryngoscopic view showing an irregular elevated lesion at the flap margin. (b) FDG-PET image demonstrating focal uptake corresponding to the recurrent lesion.

The patient was a 67-year-old man with no remarkable medical or family history. He had a 25-year history of smoking 40 cigarettes per day. Two years prior, he had undergone surgery for left buccal mucosal squamous cell carcinoma (cT3N0M0, stage III). At one year and 11 months after surgery, local recurrence was identified in the caudal portion of the reconstructive skin flap, and he was referred to our department for cisplatin-based chemoradiation therapy. At the time of the presentation, his performance status was 0, and he was asymptomatic with a generally good condition. Imaging studies revealed that the recurrent lesion was confined to the caudal portion of the left buccal mucosal flap. Following deliberation with the surgical team, the decision was taken that prophylactic neck irradiation was not required, given the prior neck dissection and the fact that this represented a purely local recurrence 1 year and 11 months after surgery. Because the radiation field was limited to this area, he was considered a suitable candidate for intraoral device therapy using BinkieRT (Figure 1).

This study was reviewed and approved by the ethics review board of our institution (Approval Number: 2171). Written informed consent was obtained from the patient for publication of this report and the accompanying images.

**Figure 2:**
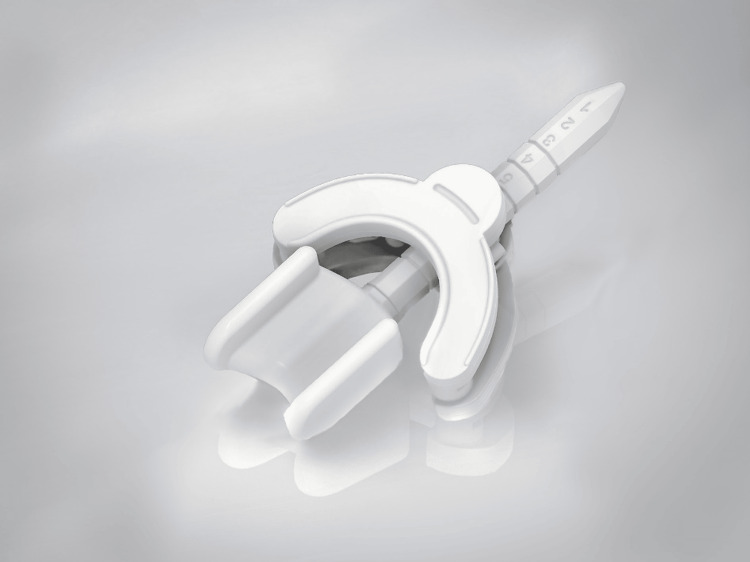
A tongue positioning device (BinkieRT Type C) featuring a C-shaped paddle blade. (width 26 mm, depth 87.8 mm). Permission to reproduce this image was obtained from Paprica Lab [7].

A C-type BinkieRT was used to displace the tongue toward the right, opposite the left buccal mucosa (Figure 2).

Planning CT scans were acquired both with and without the device, with thermoplastic mask immobilization in each case. Target contouring was performed using the Monaco® version 5.11.03 treatment planning system (Elekta AB, Stockholm, Sweden). The gross tumor volume (GTV) was delineated based on flexible nasopharyngeal laryngoscopy, MRI, and FDG-PET findings. The clinical target volume (CTV) was defined as the GTV plus a margin of 5-10 mm, excluding anatomical barriers. The device was positioned between the buccal mucosa and the tongue, allowing clearer delineation of these structures and improved CTV definition. Irradiation was confined to the local site with daily CBCT-based image guidance. Furthermore, the BinkieRT device ensured consistent compression and stabilization of the buccal mucosa, thereby minimizing intra-fractional internal motion. Consequently, the planning target volume (PTV) margin was reduced from 3 mm (without the device) to 2 mm (with the device). The tongue was delineated as an organ at risk (OAR), with its inferior border defined at the level of the superior margin of the hyoid bone. Although tongue morphology differed between the two CT sets, the total contoured tongue volumes were approximately equivalent. An IMRT plan was created using the Precision® version 3.3.1.3 planning system (Accuray Inc., Sunnyvale, CA, USA). The prescribed dose was 70 Gy in 35 fractions. The plan ensured that 95% of the PTV received 100% of the prescribed dose (D95%) while minimizing the tongue OAR dose, and that the PTV volume receiving ≥105% of the prescribed dose did not exceed 1 cm³. Treatment was delivered using TomoTherapy® (Accuray Inc.) with 6 MV X-rays.

**Figure 3:**
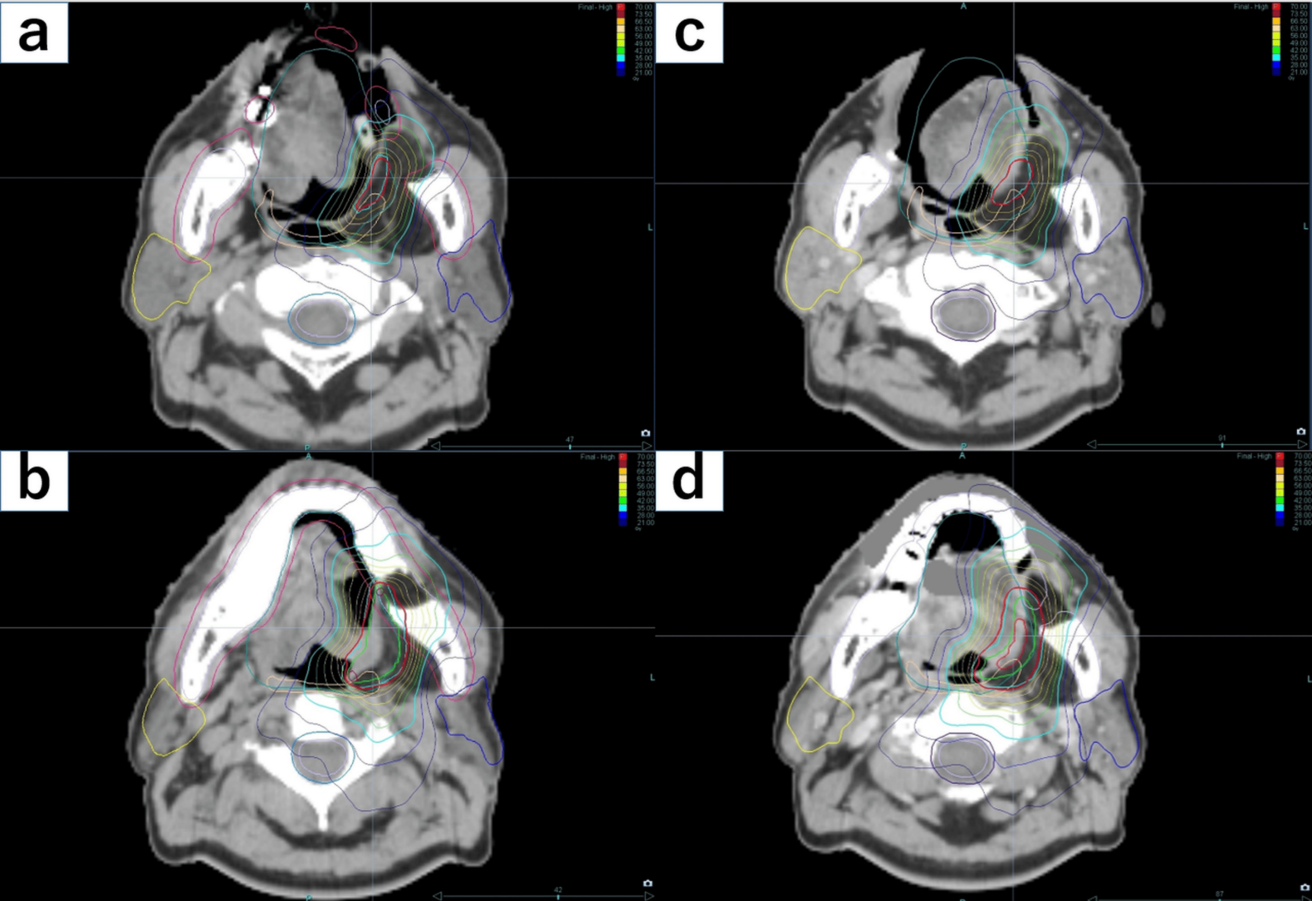
Axial dose distributions (a)(b) with and (c)(d) without the BinkieRT device. Use of the BinkieRT device effectively reduced the tongue dose by displacing the tongue away from the target volume.

Dose distributions with and without the device are shown in Figure 3.

**Figure 4:**
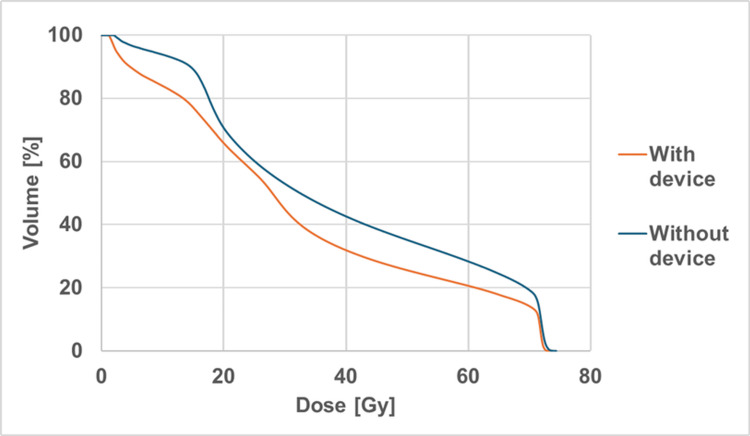
A comparison of DVH parameters of the tongue in IMRT plans with and without the BinkieRT device.

DVH parameters for the tongue OAR, including the percentage of volume receiving 15, 30, 45, 60, and 70 Gy (V15-V70), maximum dose (Dmax), and mean dose (Dmean), are summarized in Table 1 and Figure 4.

**Table 1:** A comparison of tongue dosimetric parameters (V15Gy to V70Gy, Dmax, Dmean) in IMRT plans with and without the BinkieRT device. V15–V70: the percentage of volume receiving 15, 30, 45, 60, and 70 Gy. Dmax: maximum dose. Dmean: mean dose.

Parameters	With device	Without device
V15Gy（%）	72.9	88.2
V30Gy（%）	38.8	52.5
V45Gy（%）	26.8	38.4
V60Gy（%）	20.0	28.3
V70Gy（%）	14.3	19.5
Dmax（Gy）	73.89	74.83
Dmean（Gy）	31.38	38.79

DVH analysis demonstrated reductions in V15-V70 and Dmean with the device, whereas Dmax was comparable between the two plans. The observed dose reduction was likely attributable to tongue displacement away from the irradiation field, improved visualization of the buccal mucosa-tongue interface, and a smaller PTV margin due to enhanced setup accuracy.

**Figure 5:**
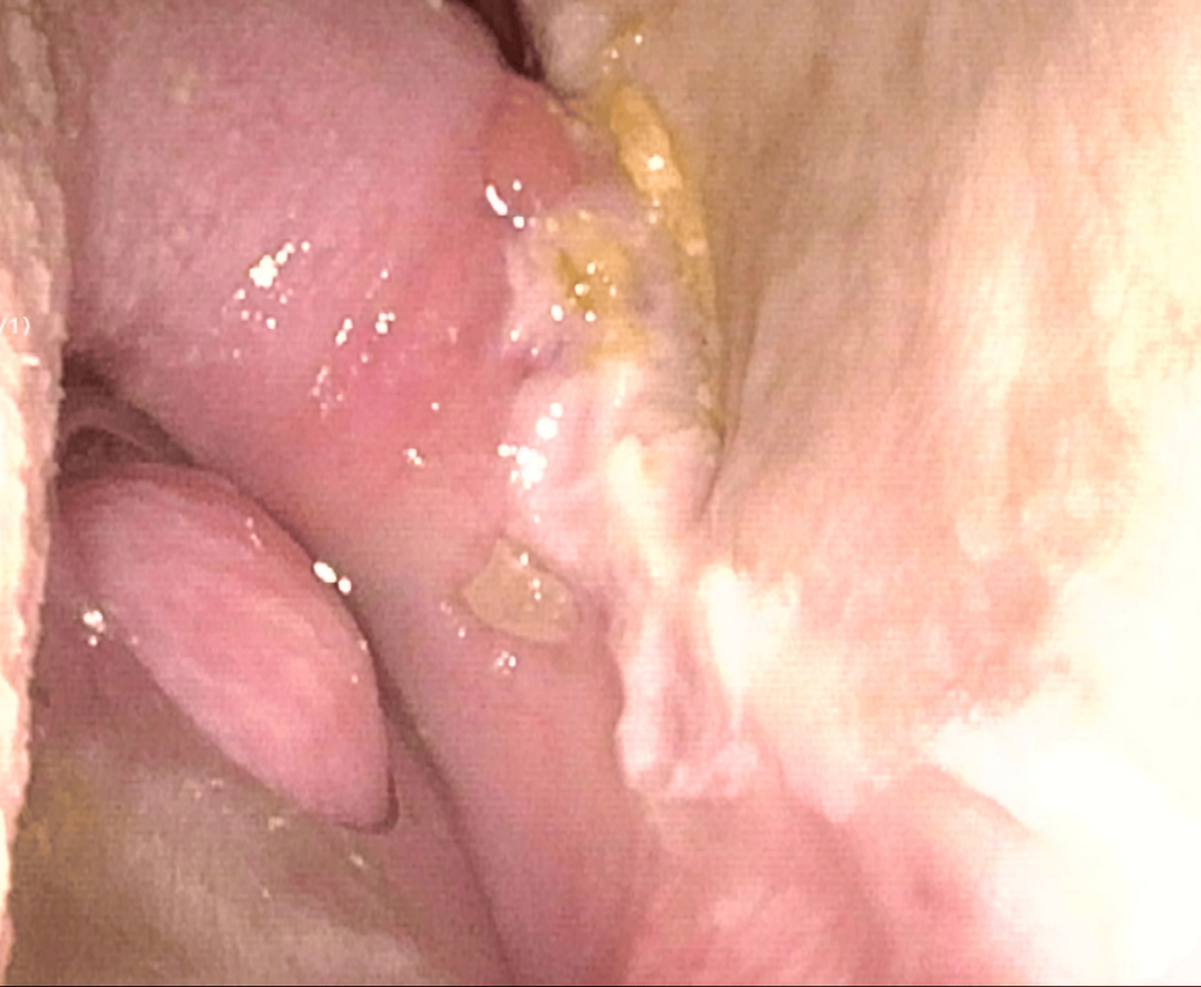
Flexible nasopharyngeal laryngoscopy at two months post-treatment showing a near-complete response of the tumor.

The actual treatment was administered using a plan created with the BinkieRT device in place. According to the Common Terminology Criteria for Adverse Events (CTCAE) v6.0, grade 1 oral mucositis developed on day 21 at a cumulative dose of 28 Gy, followed by grade 2 dysgeusia and grade 1 xerostomia on day 42 at a cumulative dose of 58 Gy. Although the patient reported mild pain associated with mucositis, he continued to tolerate the intraoral device without difficulty, and radiotherapy was completed as planned without interruption. Two months after treatment completion, mucositis had fully resolved. Despite the persistence of mild dysgeusia and xerostomia, he was able to maintain oral intake comparable to his pretreatment level. Flexible nasopharyngeal laryngoscopy at that time confirmed a near-complete macroscopic response of the tumor (Figure 5).

## Discussion

This case demonstrates that the tongue positioning device BinkieRT can substantially reduce radiation dose to the tongue and oral cavity during IMRT for oral cancer. Dose reduction to oral OARs is clinically important because radiation-induced toxicities such as mucositis, dysphagia, and dysgeusia occur in a dose-dependent manner [1-3]. Therefore, minimizing the dose to these structures may contribute to preserving patients’ quality of life.

Several prior studies have reported the benefits of tongue positioning for OAR sparing. Kil et al. showed significant reductions in tongue dose with a “stick-out tongue” position [8], and Ikawa et al. demonstrated that a tongue depressor-equipped mouthpiece helped prevent lingual mucositis [5]. Hong et al. further described a semi-custom intraoral device fabricated using a 3D printer [6]. Compared to these devices, BinkieRT is highly practical due to its ease of use and its capacity to effectively reduce radiation exposure to the tongue and oral cavity.

While X-ray-based IMRT has limitations in reducing doses to OARs and cannot achieve the superior dose sparing of intensity-modulated proton therapy (IMPT) [9,10], the clinical availability of IMPT remains limited due to the small number of facilities capable of delivering it. The combination of X-ray-based IMRT with a simple intraoral device such as BinkieRT may provide a more accessible, cost-effective, and practical means of dose reduction.

Previous reports have described intraoral devices that require 3D printing or dental intervention, which can be time-consuming and technically demanding [11,12]. In contrast, BinkieRT requires no special preparation and can be implemented immediately in daily clinical practice. Furthermore, its fixation accuracy and reproducibility have already been validated by a Korean group [13], supporting its reliability for routine use.

The limitation of this report includes its single-case nature, which precludes assessment of long-term outcomes or late toxicities. Further accumulation of clinical cases and prospective evaluations is warranted to confirm the efficacy and safety of BinkieRT-assisted RT.

## Conclusions

BinkieRT facilitated noninvasive, immediate reduction of radiation dose to the tongue and oral cavity during IMRT for oral cancer. This may help prevent radiation-induced oral toxicities and maintain patients’ quality of life. BinkieRT requires no special skills and can be implemented immediately, thereby possibly serving as a simple and practical adjunctive tool for daily clinical use.
